# Discovery of Hit Compounds Targeting the P4 Allosteric Site of K-RAS, Identified through Ensemble-Based Virtual Screening

**DOI:** 10.1021/acs.jcim.3c01212

**Published:** 2023-10-12

**Authors:** Patricia Gomez-Gutierrez, Jaime Rubio-Martinez, Juan J. Perez

**Affiliations:** † Department of Chemical Engineering. ETSEIB, Universitat Politecnica de Catalunya, Av. Diagonal, 647, Barcelona 08028, Spain; ‡ Allinky Biopharma, Madrid Scientific Park, Faraday, 7, Madrid 28049, Spain; § Department of Materials Science and Physical Chemistry, University of Barcelona and the Institut de Recerca en Quimica Teorica i Computacional (IQTCUB), Marti i Franques, 1, Barcelona 08028, Spain

## Abstract

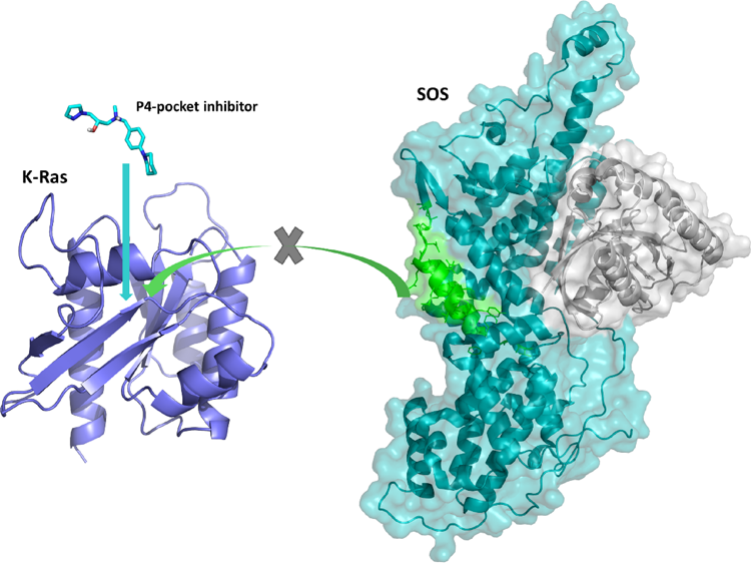

Mutants of Ras are oncogenic drivers of a large number of human tumors. Despite being recognized as an attractive target for the treatment of cancer, the high affinity for its substrate tagged the protein as undruggable for a few years. The identification of cryptic pockets on the protein surface gave the opportunity to identify molecules capable of acting as allosteric modulators. Several molecules were disclosed in recent years, with sotorasib and adagrasib already approved for clinical use. The present study makes use of computational methods to characterize eight prospective allosteric pockets (P1–P8) in K-Ras, four of which (P1–P4) were previously characterized in the literature. The present study also describes the results of a virtual screening study focused on the discovery of hit compounds, binders of the P4 site that can be considered as peptidomimetics of a fragment of the SOS αI helix, a guanine exchange factor of Ras. After a detailed description of the computational procedure followed, we disclose five hit compounds, prospective binders of the P4 allosteric site that exhibit an inhibitory capability higher than 30% in a cell proliferation assay at 50 μM.

## 1 Introduction

The Ras family of proteins represents a group of small GTPases that function as molecular signal switches, cycling between the GDP-bound inactive and the GTP-bound active forms. The two forms exhibit different conformations, granting the protein the ability to recognize diverse substrates. Two types of proteins facilitate the exchange between the active and inactive forms. On the one hand, guanine exchange factors (GEFs) catalyze nucleotide exchange from GDP to GTP, and on the other hand, GTPase activating proteins (GAPs) facilitate the hydrolysis of the γ-phosphate of the bound GTP.^1^ The Ras family comprises different protein isoforms that share a high sequence identity including H-Ras, N-Ras, K-Ras4A, and K-Ras4B, the latter being two splicing isoforms of the same gene.^2^

This family of proteins engages and activates diverse downstream signaling pathways regulating different cellular responses, including proliferation, survival, and differentiation.^3,4^ Specifically, the Ras–Raf–MEK–ERK pathway controls cell proliferation by modulating the levels of diverse cell cycle regulators. Alternately, Ras can also promote proliferation and cell survival through the Ras–PI3K–Akt signaling pathway. In addition, Ras can activate other proteins like the Ral guanine nucleotide dissociation stimulator (RalGDS) or the T-cell lymphoma invasion and metastasis 1 (TIAM1), involved in the regulation of vesicle trafficking and cytoskeletal organization, respectively.^5,6^ Constitutive activation of Ras is the cause of severe pathologies including cancer.^7^ Actually, about 30% of human cancers are due to Ras constitutive activation, with the highest incidence in lung, colon, and pancreatic cancers.^1^ Dysregulation of Ras is mainly due to the occurrence of point mutations, most frequently in residues G12, G13, and Q61 that cover ca. 98% of all mutations associated with Ras,^1^ with K-Ras being the predominantly mutated isoform (85%).^8^ These mutations leave the protein in a constitutively active state due to a decreased intrinsic GTPase activity and/or to an increased resistance to GAP-mediated hydrolysis^1,9^ resulting in aberrant signaling.

Recognized as an attractive target for the treatment of cancer, the high cytosolic concentrations of GTP and GDP, together with their high affinity for Ras, rendered the design of competitive inhibitors elusive.^10^ Moreover, the lack of obvious alternative pockets in its crystal structure made the protein to be considered for a long time as undruggable.^11^ However, analysis of the protein dynamical behavior carried out by means of computational studies revealed the existence of transient pockets susceptible to be exploited for the design of allosteric inhibitors.^12,13^ As a proof of concept, diverse low-affinity inhibitors were subsequently identified and reported in the literature.^14−18^ Today, sotorasib^19^ and adagrasib,^20^ allosteric inhibitors specifically targeting the G12C mutant, are already approved for clinical use.

The structure of K-Ras consists of a globular GTPase domain (residues 1–166) and a hypervariable region (167–188). Furthermore, the globular domain is composed of two lobes including the N-terminal or effector lobe (residues 1–86), where all the downstream effectors and other major regulators such as GEF and GAP proteins make major contacts,^21^ and the C-terminal or allosteric lobe (residues 87–166) engaged in dimerization.^22^ Diverse allosteric sites were identified from computational studies,^12,13^ with four of them (P1–P4) subsequently validated experimentally.^23,24^ The P1 site or switch I/II pocket is located in the effector lobe between the β1−β3 strands and the α2 helix. Most of the allosteric inhibitors disclosed to date bind to this site including compounds described by Fesik’s group,^14^ Genetech,^15^ Kobe0065,^16^ 0375-0604,^25^ Gorfe’s group,^26^ BI-2852,^27^ or Rabbitts’s group.^28,29^ The P2 site or switch II pocket is also located on the effector lobe between helices α2 and α3 and the P-loop. This is the binding site of irreversible inhibitors targeting the G12C K-Ras mutant like sotorasib,^19^ adagrasib,^20^ or ARS-853.^30^ Moreover, recent reports suggest that inhibition of K-Ras through this site can be achieved beyond the G12C mutants, opening the door for designing more general therapeutic agents.^31^ In this direction, a reversible ligand (TH-Z835) targeting the G12D mutant^32^ and a reversible pan K-Ras compound (BI-2865) were recently disclosed.^33^ P3 is located in the allosteric lobe between the C-terminus of helix α3, the L7 loop, and the C-terminus of helix α5. Compound KAL-21404358 is one of the few compounds disclosed shown to bind to this site.^34^ Interestingly, the compound Zn^2+^-cyclen binds with low affinity to a P3 subpocket termed P3b.^35^ Both compounds favor the state 1 versus the state 2 of the active, GTP bound K-Ras structure, characterized by the loss of hydrogen bonding interactions between the γ-phosphate and residues Thr^35^ and Gly^60^ and associated with a weaker affinity to effector molecules.^34−36^ Finally, the P4 site is located between the effector and the allosteric lobe close to the nucleotide binding site. It involves residues of the switch I, the α1 helix, and β2 and emerges when the switch I adopts an open conformation bound to GDP. Located at the interaction interface of some downstream effectors such as Raf, PI3K, or RalGDS,^37^ there is no definitively recognized small molecule binder yet. Andrographolide and its derivatives SRJ09 and SRJ23 were proposed to bind the site,^38^ although subsequent studies point to the switch I/II pocket as the most likely spot.^39^

Druggability of K-Ras by inhibition through the P4 site is puzzling because there is no direct evidence of a small molecule binder, although the antibody mimetic DARPin K27 binds to the site and inhibits K-Ras downstream signaling.^40^ This raises the question of whether the site is affordable only to macromolecule ligands. Aimed at exploring the viability of the site for designing novel small molecule K-Ras inhibitors, we report in the present work the results of a virtual screening study on the site and disclose novel hit molecules as prospective allosteric modulators. For this purpose, we first carried out a study of the dynamic behavior of the protein using molecular dynamics to better characterize the structural features of the P4 site. These results permitted defining a query that was used for screening the ZINC chemical library.^41^ As a result, we conclude this report by disclosing the structures of five molecular hits, prospective binders of the P4 site.

## 2 Methods

### Identification of Transient Pockets in K-Ras

The computational procedure followed to identify transient sites on the protein is explained elsewhere.^42^ Specifically, protein was subjected to a 500 ns accelerated molecular dynamics (aMD) calculation within the classical (NVT) ensemble using the protocol implemented in Amber12.^43^ The solvent was treated explicitly using the TIP3P water model, and periodic boundary conditions were applied. Energy was computed using the Amber ff99SB force field,^44^ although parameters for GDP were taken from an alternative source.^45^ A cutoff distance of 10 Å was set for short-range noncovalent interactions, whereas electrostatic interactions were treated using the PME method.^46^ The temperature was kept constant at 300 K by means of a Langevin thermostat.^47^

The simulation was carried out on the structure of the human K-RasG12 V bound to GDP solved at 1.76 Å resolution (PDB ID: 4EPX),^14^ whose atomic coordinates were retrieved from the RCSB PDB website.^48^ Protonation states of the diverse residues were assigned using the Protonate3D function of the MOE program.^49^ The structure was soaked in a rectangular box of 12,876 water molecules, to which six Na^+^ ions were added to maintain the neutrality of the system. The system was energy minimized to relax the initial structure in several steps using the steepest descent method. In the first step, only side chains were free to move, applying to the protein backbone atoms and GDP-Mg a positional constraining harmonic potential with a force constant of 1 kcal/mol Å^2^. In the following steps, restrictions were gradually lifted, first on the protein backbone and then on the GDP-Mg system. Next, the temperature of the system was gradually increased using 100 ps of NVT molecular dynamics at a rate of 30 K every 10 ps. Subsequently, the system was subjected to an equilibration process consisting of 1 ns of molecular dynamics in the NPT ensemble and 1 ns of molecular dynamics in the NVT ensemble. The SHAKE algorithm was used to restrict the elongation motion of the bonds involving hydrogen atoms,^50^ which allowed using an integration time of 2 fs.

Conformational sampling was performed using aMD for 500 ns at 300 K. A biased potential was used in both the total and dihedral torsional energies, respectively, by adding a bias to the true potential to improve the sampling efficiency. Bias potentials were determined from the average potential energy (Ep = −12241 kcal/mol) and the average dihedral energy (Ed = 2394 kcal/mol) from a previous 50 ns classical molecular dynamics trajectory and the use of system size dependent tuning parameters αP = 8275 and αD = 120.^51^ After aMD calculations were completed, artifacts produced by the biased potential were removed using a 10th-order Maclaurin series reweighting process for each configuration to recover the canonical ensemble.^51^

Conformational analysis was carried out using 50,000 structures of the trajectory taken at regular intervals of 10 ps. Structures were aligned using the Cα coordinates of the residues included in the invariant nucleus (i.e., residues with a smaller displacement in regard to their initial position along the trajectory) to the first structure of the trajectory using the cpptraj module of the Amber12 program.^43^ To identify the diverse conformations the protein attains during the sampling process, we carried out a hierarchical cluster analysis by means of the average link algorithm^52^ using the backbone Cα root-mean-square deviation as a measure for the distance between two conformations. Subsequently, mapping of transient binding sites on the protein surface was carried out on each of the cluster representative structures using the FTMap procedure.^53^ It consists of sampling billions of positions of small organic molecules used as probes and scoring their poses using a detailed energy expression. Regions that bind several probe types serve to identify binding hot spots.

### Cryptic Pocket Characterization

The druggability of each of the prospective binding sites was assessed by means of the SiteMap program.^54^ The procedure computes a druggability score for the target site. The computation includes diverse terms that promote ligand binding including adequate size, isolation from the solvent, and penalizes increasing hydrophilicity.

### Virtual Screening

A pharmacophore was developed for any binding site of interest by means of the Site Finder program of MOE.^49^ In addition, an exclusion volume was included as restriction to disregard structures that, despite complying with the pharmacophore, may exhibit steric effects with the protein.

Virtual screening was carried out using the ZINC chemical library^41^ filtered to contain lead-like molecules only.^55^ Prior to undertaking the virtual screening process, up to 1000 conformations of each molecule were generated using the conformational import function of MOE.^49^ Molecules were first screened to determine the pharmacophore of the site of interest. Then, the compounds selected were subjected to a molecular docking process using the Glide program.^56,57^ Next, poses resulting from the docking process were filtered again for pharmacophore fulfillment using a lower degree of compliance. Finally, a diversity analysis of the set of compounds was performed by means of the canvas program^58,59^ using a molecular fingerprint that encodes the three-point pharmacophores that meet the 3D structures of the generated protein–ligand poses. Visual inspection of the complex structures permitted selecting those compounds to be procured from commercial vendors.

### *In Vitro* Tests

Cell proliferation inhibition assays were conducted using the NIH/3T3 fibroblast cell line transformed with K-RAS oncogene DNA with the G12 V mutation by transfection. Cells were cultured in DMEM supplemented with 5% fetal bovine serum (FBS). The cultured cells were seeded in tissue culture microplates at a density of 2 × 10^3^ cells/cm^2^ and incubated for 24 h at 37 °C in a humidified atmosphere containing 5% CO_2_. Test compounds were first dissolved in DMSO 100% and subsequently diluted to 50 μM, embedding a maximum 0.5% DMSO concentration, and incubated in the cell culture for another 72 h. After incubation, proliferation was quantified using the CellTiter 96 Aqueous Non-Radioactive Cell Proliferation Assay-MTS (Promega 10 #G5421) following manufacturer's instructions. Absorbance at 490 nm that is directly proportional to the number of living cells was recorded with a BMG Fluostar Optima Microplate Reader and normalized relative to control cells treated with the vehicle.

## 3 Results and Discussion

### Identification of Transient Binding Pockets in K-Ras

Transient binding pockets on K-Ras were identified from the analysis of 50,000 structures taken from the aMD trajectory at regular intervals of 10 ps. The first step consisted of classifying the diverse conformations into groups by means of cluster analysis. This required the computation of a distance matrix for all pairs of structures using the root-mean-square deviation (rmsd) of the backbone Cα atoms once the structures were previously aligned using the less flexible elements of the protein, as explained below. In the second step, FTMap^53^ was used to identify hot spots on each of the diverse clusters characterized.

**Figure 1:**
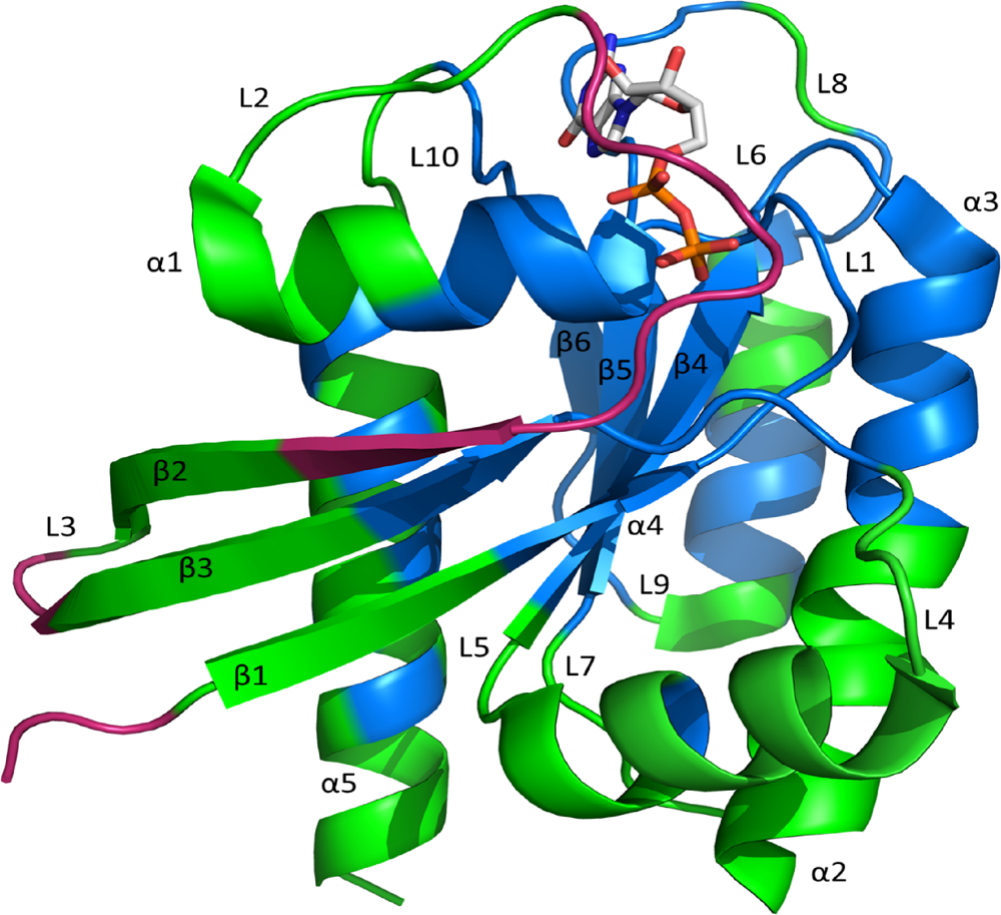
Structural elements of K-Ras and their differential flexibility. The most flexible elements are depicted in red, and the least flexible elements are in blue, whereas those showing intermediate flexibility are depicted in green.

Figure 1 shows the structure of K-Ras color-coded according to residue flexibility, and Figure S1 shows the root-mean-square fluctuations of each residue. Inspection of both figures suggests that switch I is the most flexible structural motif, together with the N-terminus of the β2-strand (red in Figure 1). In contrast, the P-loop is one of the most stable structural elements during the simulation, together with the β protein core (β4−β6), the C-termini of the β1 and β3 strands, helix α1 N-terminus, helix α3 N-terminus, and the central part of helix α4 (blue in Figure 1). The rest of the elements, including switch II and loop L7, exhibit intermediate flexibility (green in Figure 1). The set of most stable structural elements was used to superimpose the 50,000 structures and to compute a distance matrix between each pair of structures using the backbone Cα rmsd. The subsequent cluster analysis was carried out by means of the average link algorithm^52^ that permitted to group the structures into six clusters. Clusters were represented by the structure with the closest root-mean-square deviation (rmsd) to the average value of the group. Figure S2 shows the representative structures of each cluster superimposed with the crystal structure used as starting point for the MD simulations in this work (PDB ID: 4EPX).^14^ Inspection of Figure S2 suggests that the differences between structures are located on the switch I and switch II regions, in addition to specific secondary elements linked to them like the β2 strand and the α3 helix, as well as the loops L4 and L7.

**Figure 2:**
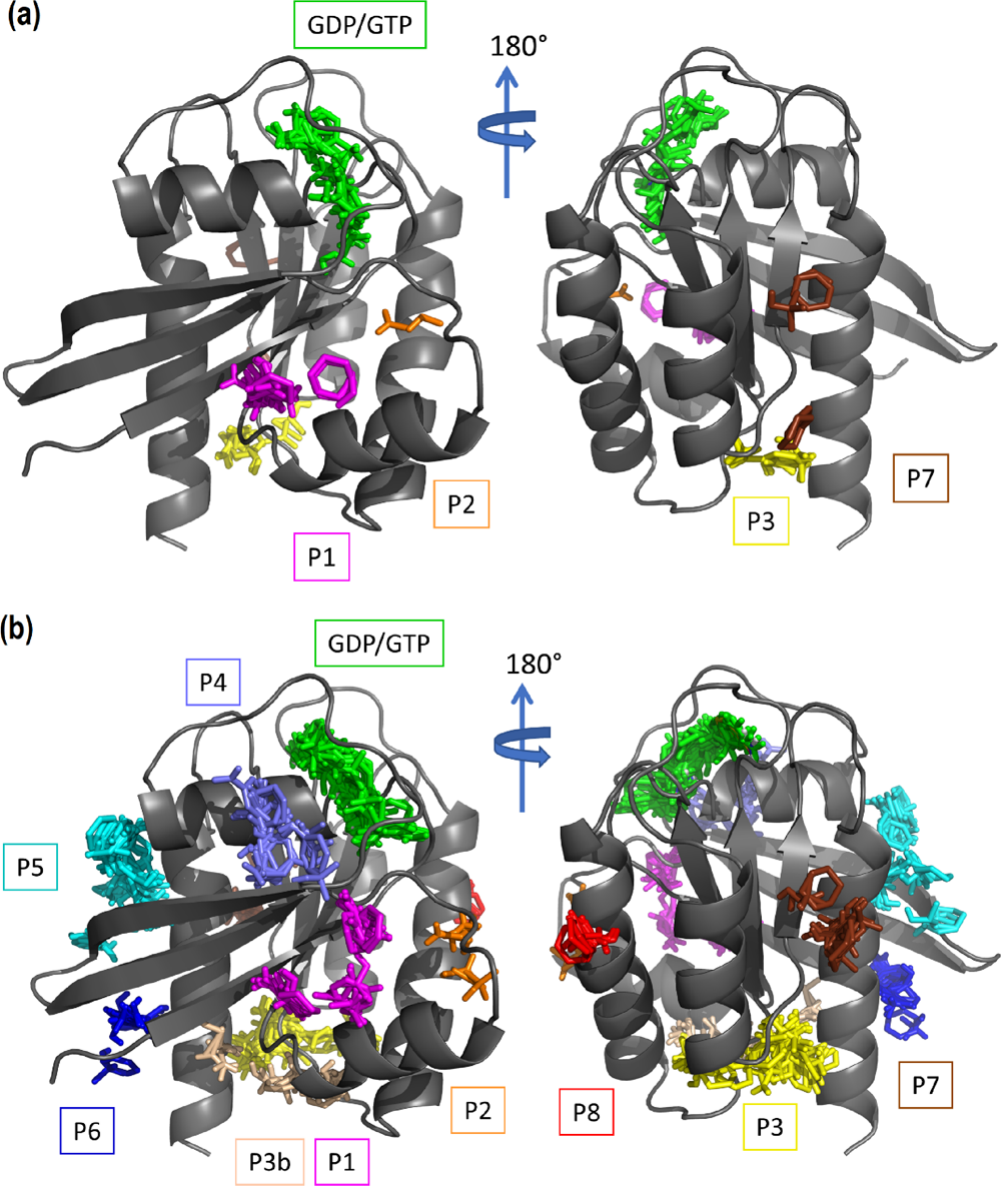
Mapping of molecular fragments from the FTMap calculations. (a) Using the crystallographic structure 4EPX as template and (b) aggregated results using each of the six cluster representatives.

Mapping hot spots on the protein surface for each of the six cluster representative structures was performed using the FTMap program.^53^ For comparison purposes, we also mapped the crystallographic structure used in the present work. The aggregated results of the mapping on each of the six representatives as well as that of the crystal structure are shown in Figure 2. As can be seen, in addition to the nucleotide-binding site, eight different sites were identified and labeled P1–P8. Sites P1–P4 correspond to those well-characterized and validated sites described above.^23,24^ The rest of the sites identified (P5–P8), despite a few of them being described previously in computational studies,^12,13,60^ are not yet experimentally validated. Interestingly, only four of these sites (P1, P2, P3, and P7) appear in both the crystal and at least one of the representative structures, stressing that binding hot spot identification requires inclusion of the dynamical behavior of the protein.

A druggability profile of the eight sites identified was investigated using SiteMap.^54^ This procedure permits classifying sites into *druggable*, *difficult*, and *undruggable* by means of a druggability score, defined as a composite index comprising diverse properties like size, exposure, enclosure, hydrophobicity, and hydrophilicity.^54^ However, the threshold of the druggability index differentiating the diverse categories is fuzzy, and although it provides an overall view, values of other properties can be used to fine-tune the classification of a specific site.^54^

Two druggability indexes were computed in the present work and are shown in the first two columns of Table 1. Specifically, the Sscore is a composite index computed using parameters like size, enclosure, and hydrophilicity. On the other hand, the Dscore is computed using parameters that promote ligand binding like adequate size or isolation from solvent but also includes a penalizing term reflecting the hydrophilicity of the site.^54^ Inspection of the first two columns of Table 1 suggests that both indexes follow the same trends with small discrepancies, despite the difference between the Dscore and the Sscore for sites P7 and P5. Inspection of Table 1 suggests that none of the sites can be considered as *druggable*, with P3, P4, P1, P6, and P2 being *difficult* and the rest *undruggable*. The present results agree with the results of a previous analysis performed on sites P1–P4.^23^

**Table 1:** Druggability Characteristics of Sites P1–P8 Computed with SiteMap and Ordered by their Sscore^a^

**site**	**Sscore**	**Dscore**	**size**	**enclosure**	**hydrophobicity**	**hydrophilicity**
P3	0.879	0.828	76	0.657	0.43	1.182
P4	0.835	0.821	53	0.699	1.258	0.867
P1	0.824	0.799	46	0.734	1.249	0.855
P6	0.804	0.774	40	0.743	1.409	0.818
P2	0.760	0.731	53	0.629	0.59	1.015
P7	0.706	0.573	39	0.670	0.406	1.278
P5	0.642	0.575	26	0.670	1.185	0.939
P8	0.501	0.514	16	0.400	0.5	0.312

a Classification as *druggable*, *difficult*, and *undruggable* is loose, but as reference, *druggable* sites exhibit average Dscore values of around 1.1, *difficult* sites exhibit average Dscore values of around 0.9, and *undruggable* sites exhibit average Dscore values lower than 0.6. However, for a more accurate assessment, values of diverse specific parameters need to be considered.

Ligands targeting these sites have a potential role as allosteric regulators or protein–protein modulators depending on their specific binding location. However, it should be borne in mind that identification of a binding transient site does not guarantee that the site is necessarily functional.^61^ Concerning sites already validated, site P1 corresponds to the switch I/II pocket described above,^14−18^ the binding site of diverse ligands.^14−16,25−29^ There are also macromolecules described to bind to the site, acting as inhibitors, like the affimer K6^62^ or the monobody JAM20.^63^ In the present study, the site is identified in five of the cluster representative structures (1, 2, 3, 5, and 6) as well as in the crystal structure. Moreover, it appears number three in Table 1, being considered as a difficult. Compounds binding the P1 site likely act as protein–protein interaction inhibitors preventing effectors to bind K-Ras or, alternatively, interfere with i-mediated GDP/GTP exchange.^23^

Site P2 corresponds to the switch II pocket, the binding site of diverse irreversible inhibitors targeting the G12C mutant^19,20,30^ ] including sotorasib and adagrasib.^19,20^ There are also macromolecules binding to the site acting as inhibitors, like the affimer K3.^62^ FTMap identifies the pocket in three of the cluster representative structures (1, 4, and 5), in addition to the crystal structure. The site appears listed as *difficult* in Table 1, giving expectations of finding noncovalent small molecule ligands for the site, in agreement with recent reports describing reversible binders to the site.^31−34^ Compounds binding to the site are thought to stabilize a non-SOS-recognizable switch II conformation and, consequently, inhibit GDP/GTP exchange.^17^

P3 is the binding site of compound KAL-21404358,^34^ as well as the Zn^2+^-cyclen in a P3b subpocket,^35^ as described above. The DARPins K13 and K19 also bind to the site.^64^ In the present study, the site is identified by FTMap in five cluster representative structures (1, 2, 4, 5, and 6), as well as in the crystal structure. However, the values listed in Table 1, together with other reports,^23^ point to the site as *undruggable* due to the low hydrobophicity indicator. The site P3b should also be considered as *undruggable* due to its small size. Binding to this site occurs preferentially in state 1 of the active Ras-GTP, providing the opportunity to stabilize the protein in this state that prevents the activation of the downstream cascade.^37^

Finally, P4 was previously proposed to be one of the prospective binding sites for andrographolide and its derivatives SRJ09 and SRJ23, together with P1.^38,39^ This uncertainty is probably due to the fact that P4 is located in the vicinity of P1. Moreover, the P4 site is the target of the antibody mimetic DARPin K27 that shows inhibition of downstream signaling.^40^ In the present work, the P4 site is identified in the cluster representative 4; however, cluster representatives 2 and 5 identify a subsite. In contrast, the site cannot be identified in the crystal structure. The difference between P4 in the structure of cluster 4 and in the structures of clusters 2 and 5 is a consequence of the conformation adopted by switch I. Whereas, in the former, it is widely open, in the latter, it adopts a partially closed form, making accessible only a subsite of P4. Interestingly, the structure of the switch I adopted in the representative of cluster 4 is very close to the conformation found in the structure of the K-Ras/SOS complex (PDB entry: 1nvv), and it is associated with an increased GDP off rate.^40,65^ The results of Table 1 suggest that the site can be considered as *difficult,* a classification that agrees with previous findings.^23^

Concerning the rest of the sites identified, P5 and P6 are located between the effector and the allosteric lobes, whereas P7 and P8 are located on the allosteric lobe. P5 is located between the helix α5 N-term, the helix α1 C-term, and the β2 strand. It appears in six cluster representatives but not in the crystal structure. The pocket was identified in previous computational studies.^13,66^ Subsequent directed mutagenesis experiments on residues Asn^26^ and Val^45^ confirmed that the pocket is part of the recognition interface of the cysteine-rich domain of the Raf protein,^67^ suggesting that binders to the pocket can act as protein–protein inhibitors of Raf. Site P6, not described previously, is located between the C-terminus of helix α5, the β1 strand, and the L3 loop. This site is identified in two cluster representative structures (4 and 6) and appears as the result of the movement of the L3 loop and the β-sheet coordinated with the movement of switch I. According to the results of Table 1, site P6 appears with similar druggability parameters as P1 that label it as *difficult*. Sites P7 and P8 were described in previous computational studies.^13,60^ Site P7 is located between the α4 helix, the N-terminal part of the α5 helix, and the β6 strand. The site was recently identified as the binding pocket of the monobody NS1^68^ as well as the affimer K69.^62^ These proteins disrupt RAS dimerization, leading to the blocking of CRAF-BRAF heterodimerization and activation. P7 is analogous to a site previously described and associated with the calmodulin binding site.^69^ In the present study, P7 is identified in four cluster representative structures (2, 3, 4, and 6), being listed in Table 1 as *undruggable* due to its small size and hydrophobicity index. Finally, the P8 site is a small pocket located between helices α3 and α4 and was identified only in the cluster representative structure 4. Although the site may be considered as *difficult* according to the values of the Sscore and Dscore listed in Table 1, it should be considered as *undruggable* due to the low hydrophobicity index.

### Virtual Screening of the P4 Site

**Figure 3:**
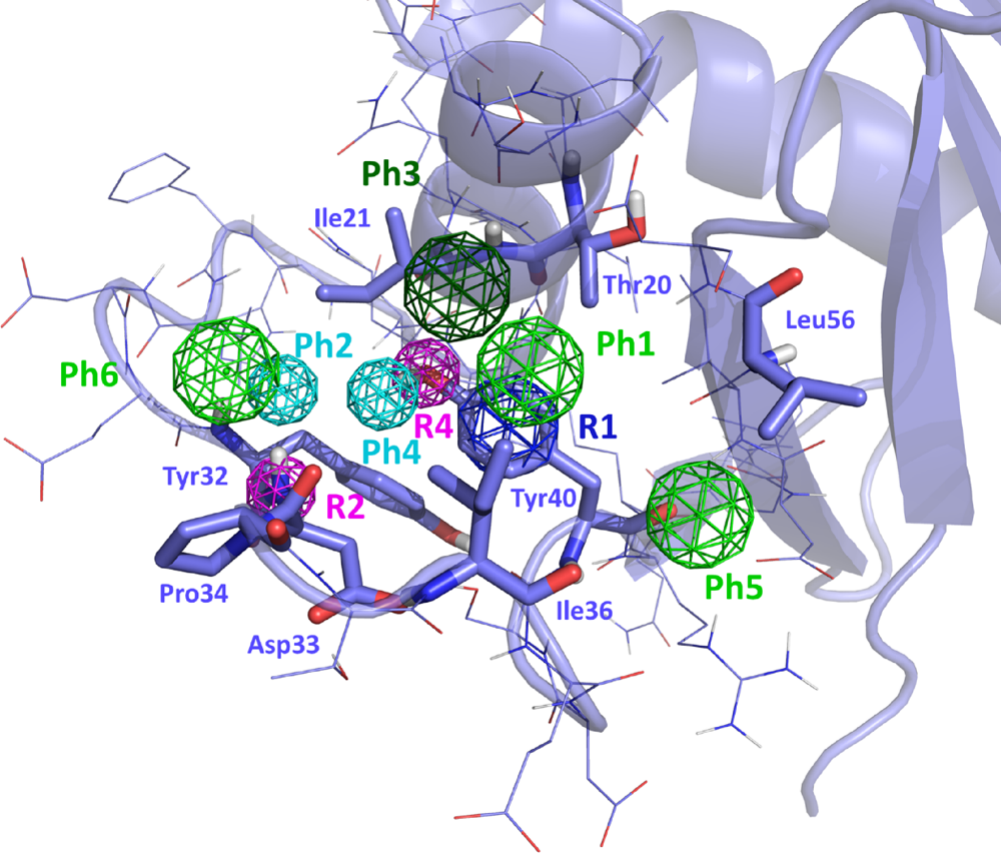
Pharmacophore of site P4. Color-coded spheres represent the features of the diverse pharmacophore points (Ph1–Ph6), as well as geometrical restrictions (R1–R3). Green represents hydrophobic/aromatic points; cyan represents proton accepting points; dark green represents hydrophobic points; navy blue represents a restriction on the aromatic ring conformation; and magenta represents a restriction regarding the direction of the proton accepting sites.

A six-point pharmacophore was defined based on the K-Ras structure using the Site Finder module of MOE.^49^ The features that binders should exhibit include (Figure 3) an aromatic ring (Ph1) located in the vicinity of Tyr^40^ and Ile^36^; two hydrogen bond acceptor points (Ph2 and Ph4) in the vicinity of the NH group of the Asp^33^ backbone and the hydroxyl group of Tyr^40^, respectively; a hydrophobic moiety (Ph3) next to Ile^21^ and Thr^20^; and two hydrophobic or aromatic moieties (Ph5 and Ph6) adjacent to residues Leu^56^ and Ile^21^, respectively. In addition, three constraints were added, two (R2 and R4) to define the direction of the hydrogen acceptor points (Ph2 and Ph4) and another to specify the orientation of the aromatic ring (R1) of point Ph1, following the PHCD pharmacophore definition of MOE.^49^ In addition, an exclusion volume that defines the binding cavity was included to eliminate structures that, despite complying with the pharmacophore, overlap their structure with that of the protein.

Screening of the ZINC compound library for pharmacophore fulfillment yielded a total of 1364 molecules and a total of 2372 conformations, with 11 of them fulfilling all restrictions. The resulting compounds were subjected to a molecular docking process using the Glide program.^56,57^ Next, to prevent the flexible docking process from taking ligands away from pharmacophore fulfillment, the diverse poses were filtered for their degree of compliance of at least six pharmacophoric points, reducing the size of the library to 336 compounds. Finally, to facilitate the selection of the final candidates, a diversity analysis was carried out using the canvas program of the Schrödinger software.^56,57^ For this purpose, we used a molecular fingerprint as a descriptor, encoding all three-point pharmacophores that meet the 3D structures of the generated protein–ligand poses. This permitted us to classify the hits into 30 clusters. Finally, a subset of 16 cluster representatives was selected according to their docking score and stereochemical complementarity with the binding site assessed by visual inspection. Of these, 13 compounds were purchased from commercial suppliers (A55001 to A55013) and submitted for *in vitro* tests.

Screening showed four compounds with a cell proliferation inhibition higher than 30% at 50 μM. This is an arbitrary cutoff to retain compounds for further investigation, loose enough not to miss any interesting hit and covering the maximal chemical diversity of the set.^70,71^ We then searched the corresponding diversity clusters of positive hits for additional compounds and selected six additional compounds for screening according to their docking score and stereochemical complementarity with the binding site, assessed by visual inspection. Four of these compounds were purchased (compounds A55014 to A55017) and submitted for *in vitro* tests, with one of them showing a cell proliferation inhibitory behavior at least 30% at 50 μM. Table 2 shows the results of the *in vitro* screening of all the compounds tested at 50 μM. Compounds A55003 and A55016 show inhibitions close to 50%, whereas compounds A55004, A55007 and A33013 inhibit cell proliferation at a 30–40% yield. The success rate of the screening process is about 30%, similar to the one observed in similar studies.^72−76^ The results shown in Table 2 reveal that the present compounds arrest proliferation. It is likely that the action is mediated through K-Ras, although it cannot be ruled out that they may enhance or activate other cellular mechanisms.

**Table 2:**
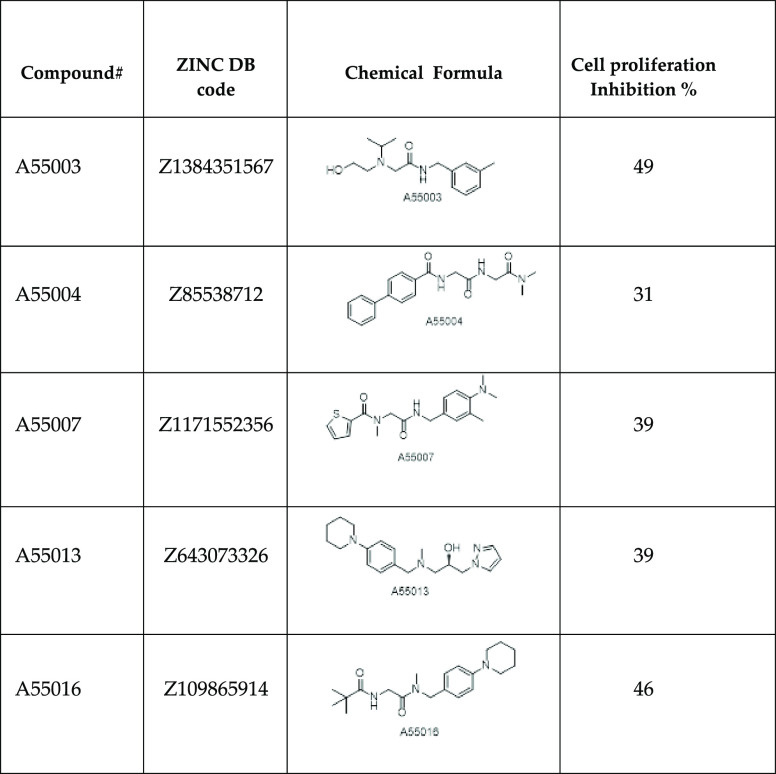
Compounds with Inhibitory Profile >30%@50 μM in a Cell Proliferation Assay^a^

a Their commercial names, chemical formula, and inhibition activity are listed in the diverse columns (raw data on cell proliferation for the diverse assays are listed in Table S1 of the Supporting Information).

**Figure 4:**
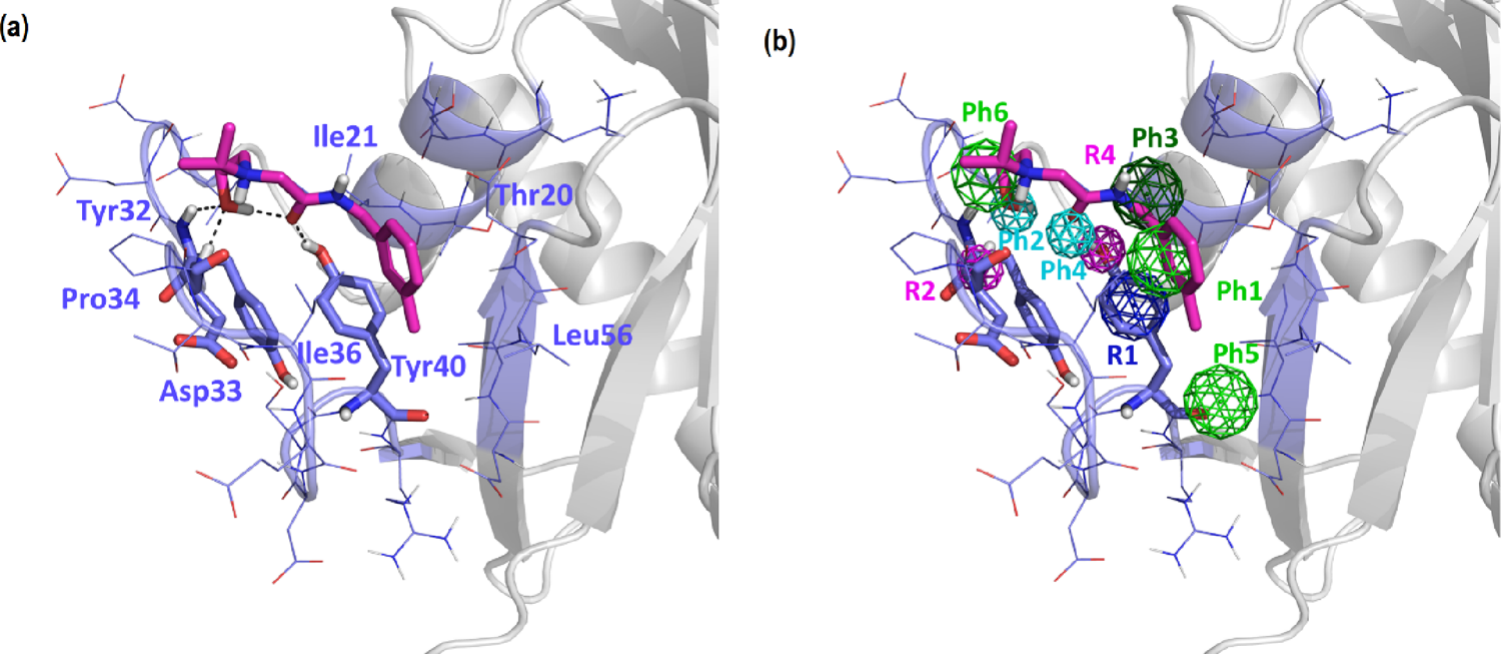
Proposed conformation of compound A55003 bound to the P4 site. (a) Bound conformation showing K-Ras residues involved and (b) fulfillment of the pharmacophore of site P4.

**Figure 5:**
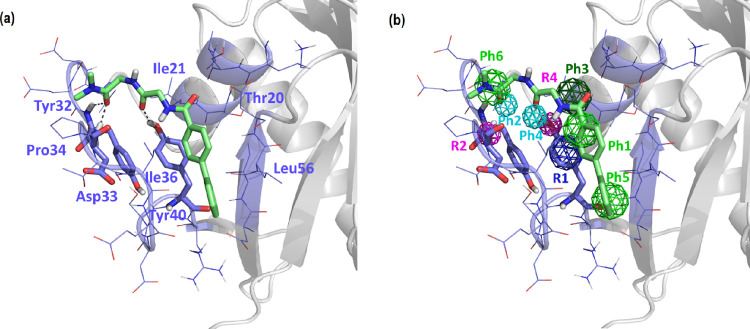
Proposed conformation of compound A55004 bound to the P4 site. (a) Bound conformation showing K-Ras residues involved and (b) fulfillment of the pharmacophore of site P4.

**Figure 6:**
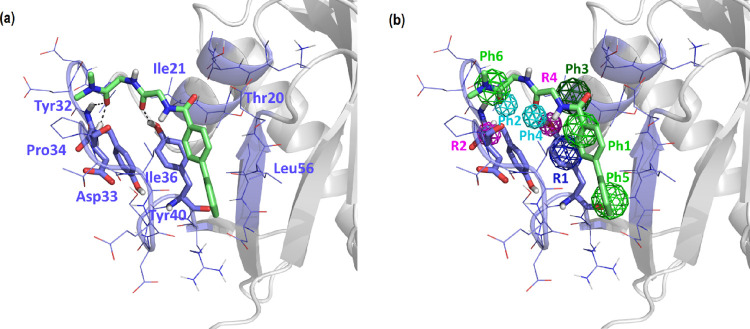
Proposed conformation of compound A55007 bound to the P4 site. (a) Bound conformation showing K-Ras residues involved and (b) fulfillment of the pharmacophore of site P4.

**Figure 7:**
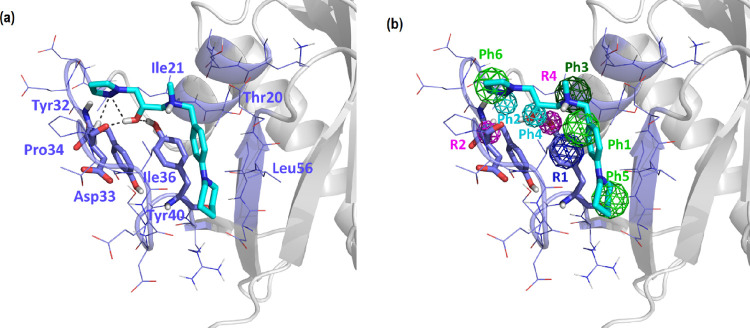
Proposed conformation of compound A550013 bound to the P4 site. (a) Bound conformation showing K-Ras residues involved and (b) fulfillment of the pharmacophore of site P4.

**Figure 8:**
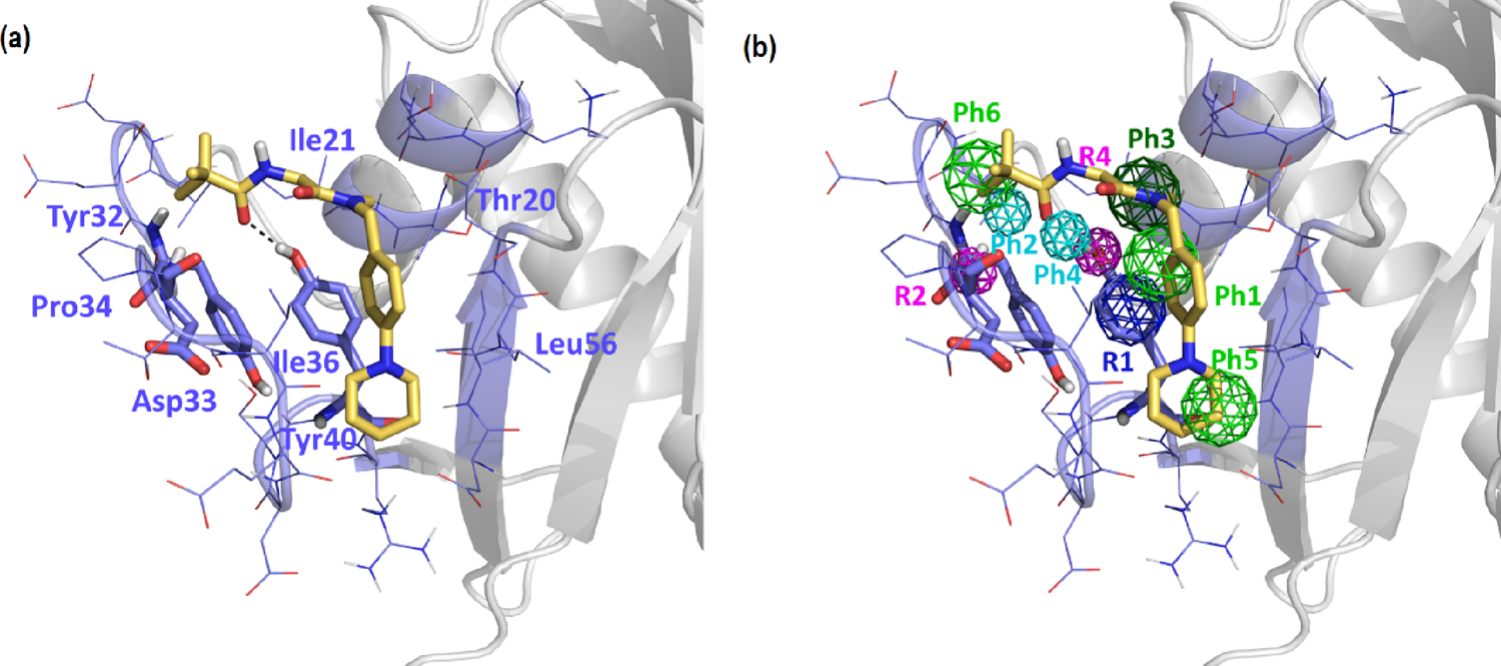
Proposed conformation of compound A550016 bound to the P4 site. (a) Bound conformation showing K-Ras residues involved and (b) fulfillment of the pharmacophore of site P4.

Figures 4–8 show the prospective bound conformations of the five compounds with cell proliferation inhibitory capacity higher than 30% at 50 μM. All the compounds fulfill pharmacophore point Ph1 by means of an aromatic ring that is placed perpendicular to the aromatic ring of Tyr^40^ and close to Ile^36^. The hydrophobic/aromatic point Ph6 is also fulfilled in all five compounds with moieties as diverse as a methyl group or a five-membered heterocyclic ring that interact with Pro^34^. Hydrophobic point Ph3 is fulfilled by means of a methyl or methylene group at a distance suitable for a van der Waals interaction with Ile^21^. All the compounds, except for A55003, fulfill the hydrophobic or aromatic pharmacophoric point Ph5. Specifically, compound A55004 fulfills the point by means of a benzene ring, compound A55007 fulfills the point by means of a methyl group, and compounds A55013 and A55016 fulfill the point by means of a piperidine ring. These moieties establish a van der Waals interaction with Leu^56^. Regarding hydrogen bond interactions, except for A55016, compounds fulfill the pharmacophoric hydrogen acceptor points Ph2 and Ph4. Specifically, compound A55003 establishes a double hydrogen bond interaction through the oxygen of an aliphatic hydroxyl group with the amide hydrogens of the Tyr^32^ and Asp^33^ backbone. The same interaction is established with the oxygen of an amide group in compound A55004, with the sulfur atom of a thiophene ring in compound A55007, and with the nitrogen in meta of the pyrazole ring of compound A55013, although the interaction is established only with the nitrogen of the Asp^33^ backbone in the latter. In contrast, the amide carbonyl of compound A55016 is far from both donor groups of the receptor to establish a hydrogen bond, despite it establishing a hydrogen bond with the hydroxyl group of Tyr^40^. All compounds, with the exception of A55013, have a central amide group that forms a hydrogen bond by means of the oxygen atom of the carbonyl group with the hydroxyl group of Tyr^40^. In contrast, compound A55013 stabilizes the interaction by means of an aliphatic-type hydroxyl group.

After analysis of the prospective bound conformations of the diverse hits, we hypothesize that binding of compounds to the P4 site may exert a stabilizing action on switch I that facilitates its open conformation. This conformation is similar to the one adopted by K-Ras when bound to SOS, more widely open than that shown in the structures of Ras-GTP in state 1 and associated with an increased GDP dissociation rate. The fact that this type of open conformation was sampled during the simulation process suggests that the recognition between Ras and SOS could respond to a conformational selection mechanism. However, the two conformations are not identical showing some differences in the arrangement of diverse residues. Accordingly, a second step of induced fit is required between the interfaces of both proteins in the recognition process.

**Figure 9:**
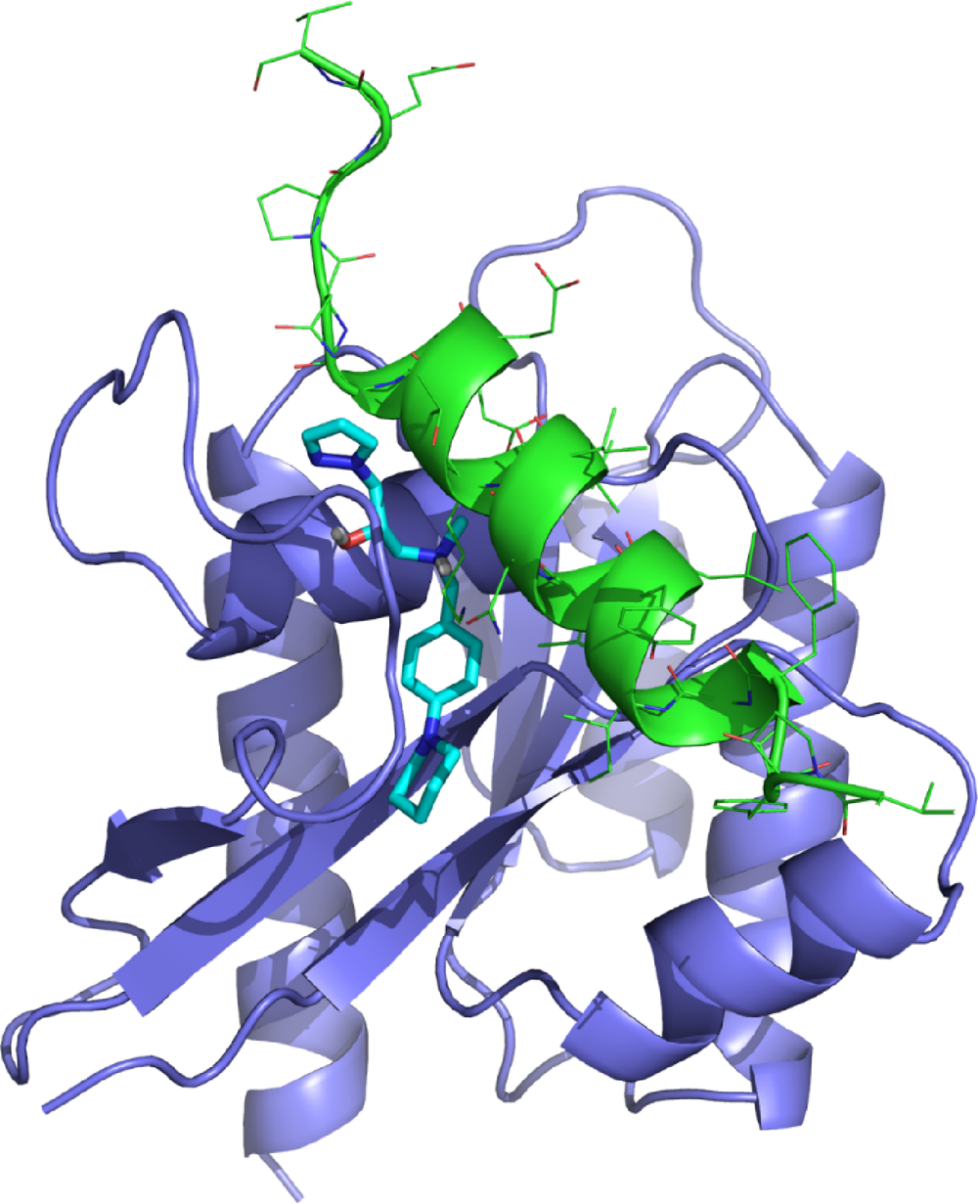
Superimposition of the SOS αI helix (green) and compound A550013 (cyan) bound to K-Ras (purple).

The compounds identified in the present work can be considered as peptidomimetics of the SOS helix αI^77,78^ interfering with the recognition between K-Ras and SOS and consequently arresting the GDP dissociation process. Inspection of the bound conformation of the active compounds showed that these compounds share a few interactions with the K-Ras/SOS complex. Specifically, all the compounds occupy in part the area of the SOS αI helix C-terminus in the K-Ras/SOS complex (Figure 9). Moreover, the interaction between their corresponding aromatic rings and Tyr^40^ in K-Ras mimics the interaction between Tyr^40^ and SOS His^911^ found in the K-Ras/SOS complex. Another interaction common to all compounds is the presence of an acceptor group forming a hydrogen bond with the backbone NH amide of Tyr^32^ and mimicking the interaction between Tyr^32^ and SOS Asn^944^ in the K-Ras/SOS complex.

## Conclusions

The present work reports the results of a modeling study aimed at identifying transient pockets of K-Ras as prospective allosteric sites to modulate protein activity. For this purpose, the dynamical behavior of the protein was investigated by means of a 500 ns molecular dynamics trajectory at 300 K using accelerated molecular dynamics as the sampling technique. Cluster analysis of the trajectory permitted us to identify six different conformations adopted by the protein. Each of the conformations was mapped for hot spots using diverse molecular probes by means of the FTMap program. The results permitted identification of eight cryptic pockets. Seven of them were previously described, with four of them already validated. We also analyzed the characteristics of the pockets.

Moreover, a virtual screening process was conducted on site P4 using the ZINC database. After a hierarchical process, we ended up with a short list of 16 compounds, 13 of which were purchased from commercial suppliers and submitted for *in vitro* testing. Four compounds exhibited cell proliferation inhibitory activity higher than 30% at 50 μM. In the second step, we selected an additional six compounds from the clusters of the positive hits for screening. Four of these compounds were purchased and submitted to *in vitro* screening. Only one of them showed cell proliferation inhibitory activity. Accordingly, the present work discloses five molecular hits with cell proliferation inhibitory activity higher than 30% at 50 μM, targeting the P4 site of K-Ras. Further studies need to be carried out to validate binding to the site and to assess their role as allosteric modulators of the K-Ras activity.

## Data Availability

MD trajectories produced during the execution of this work can be obtained from the authors upon request. In-house scripts, input files, topologies, and PDB structures of all cluster representatives used for the analysis are placed in the public repository Github: https://github.com/JaimeRubioMartinez/KRas_P4_AllostericSite.

## Supporting Information Available

Detailed results regarding the root-mean-square fluctuations per residue and the structures in PDB format of the representatives obtained from the cluster analysis (PDF)
