# Supplementary material for: Discovery of Hit Compounds Targeting the P4 Allosteric Site of K-RAS, Identified through Ensemble-Based Virtual Screening
Source: J Chem Inf Model. 2023 Oct 12;63(20):6412–22. doi: 10.1021/acs.jcim.3c01212 (PMC10598794; doi:10.1021/acs.jcim.3c01212)
Supplement: Supplementary file 1 — ci3c01212_si_001.pdf [file ci3c01212_si_001.pdf]

Supporting Information

to

Discovery of Hit Compounds Targeting the P4 Allosteric Site of K-RAS, Identified through Ensemble-Based Virtual Screening

Patricia Gomez-Gutierrez.[1,2] Jaime Rubio-Martinez[3] and Juan J. Perez[1,*]

[1]Department of Chemical Engineering. ETSEIB. Universitat Politecnica de Catalunya. Av. Diagonal. 647. 08028 Barcelona. Spain

[2]Allinky Biopharma. Madrid Scientific Park. Faraday. 7. 28049 Madrid. Spain

[3]Department of Materials Science and Physical Chemistry. University of Barcelona and the Institut de Recerca en Quimica Teorica i Computacional (IQTCUB). Marti i Franques. 1. 08028. Barcelona. Spain

Corresponding author's e-mail: juan.jesus.perez@upc.edu

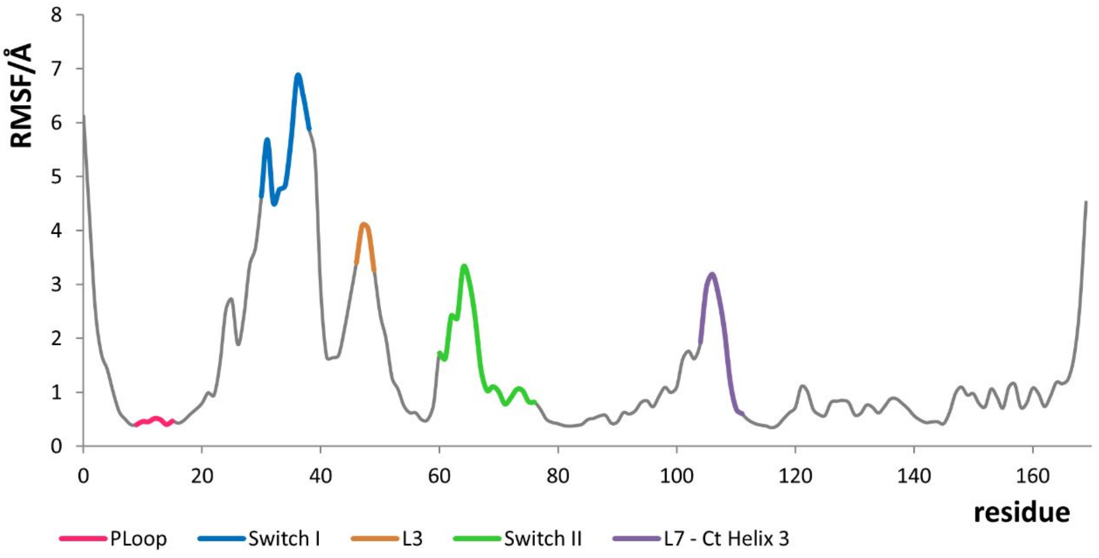

Figure S1. Root mean square fluctuations of the K-Ras Cα atoms during the aMD trajectory. Specific K-Ras structural elements are highlighted in color.

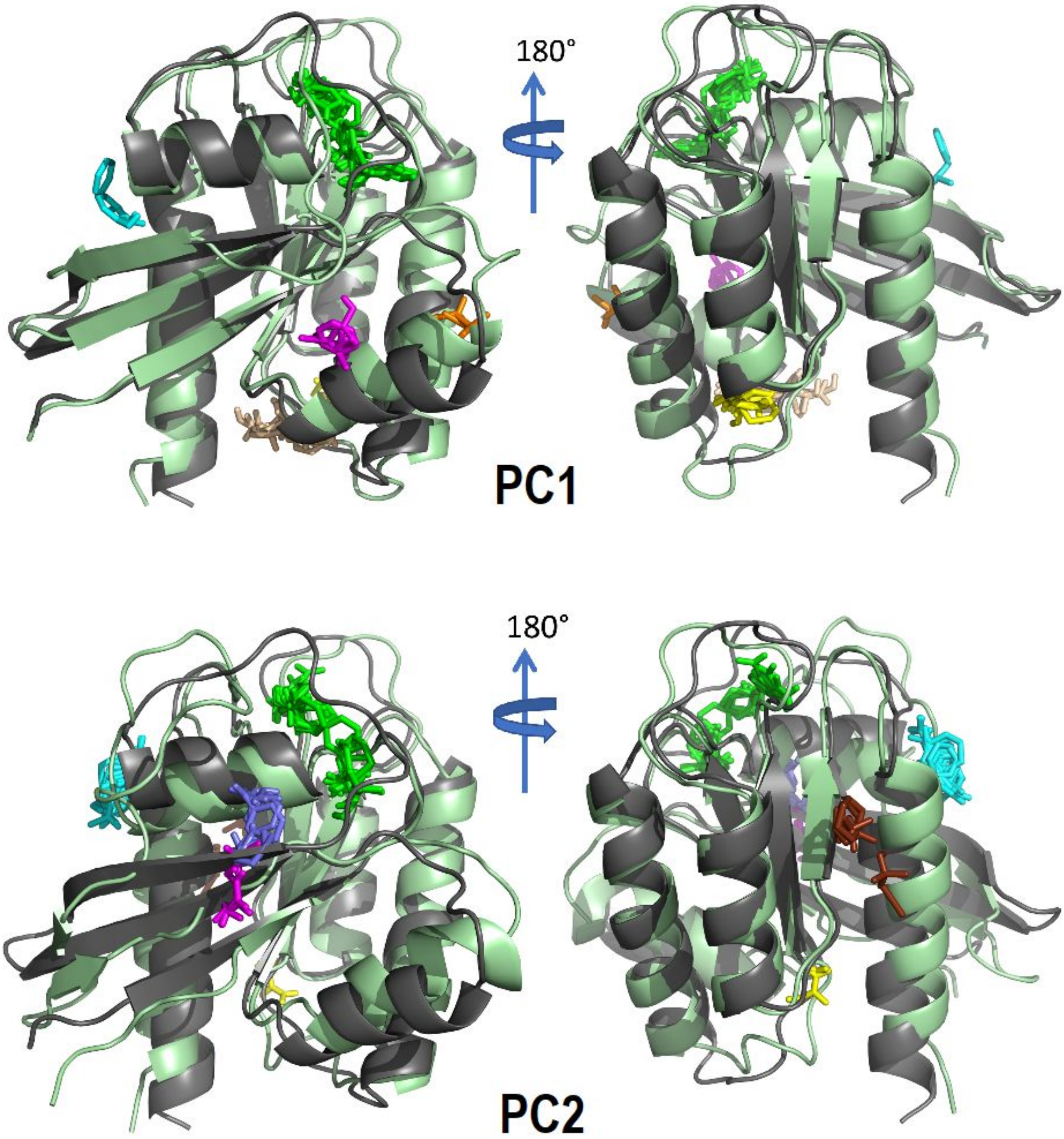
180°
PC1
180°
PC2

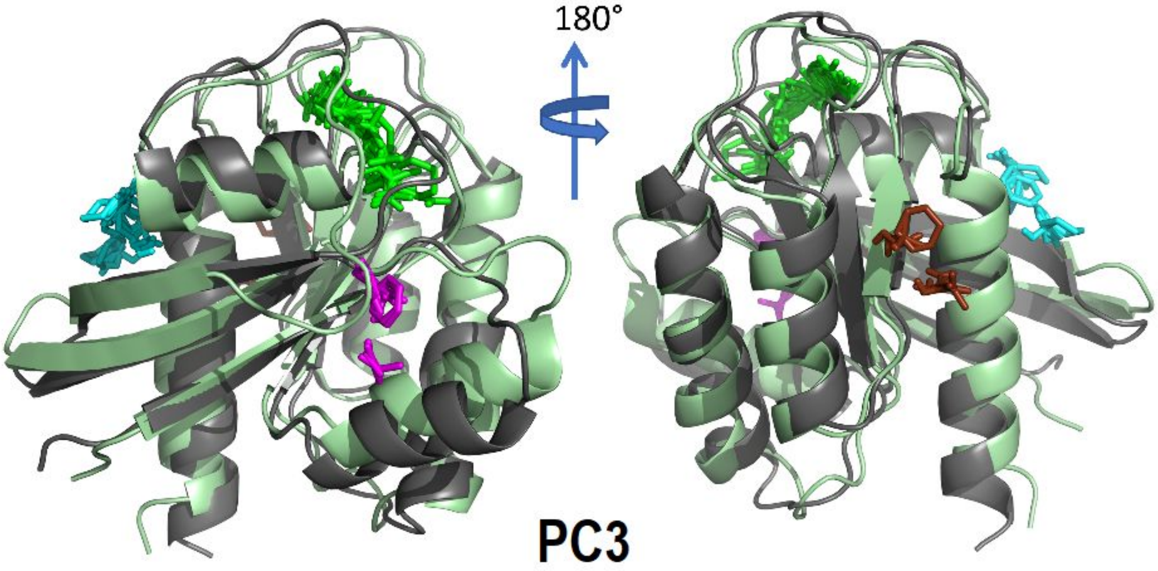
180°
PC3

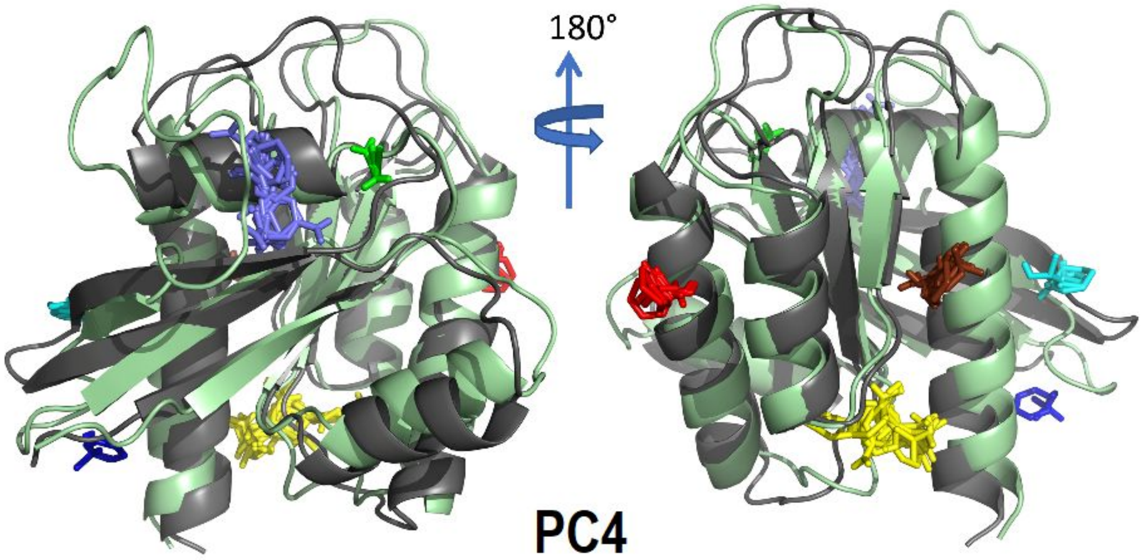
180°
PC4

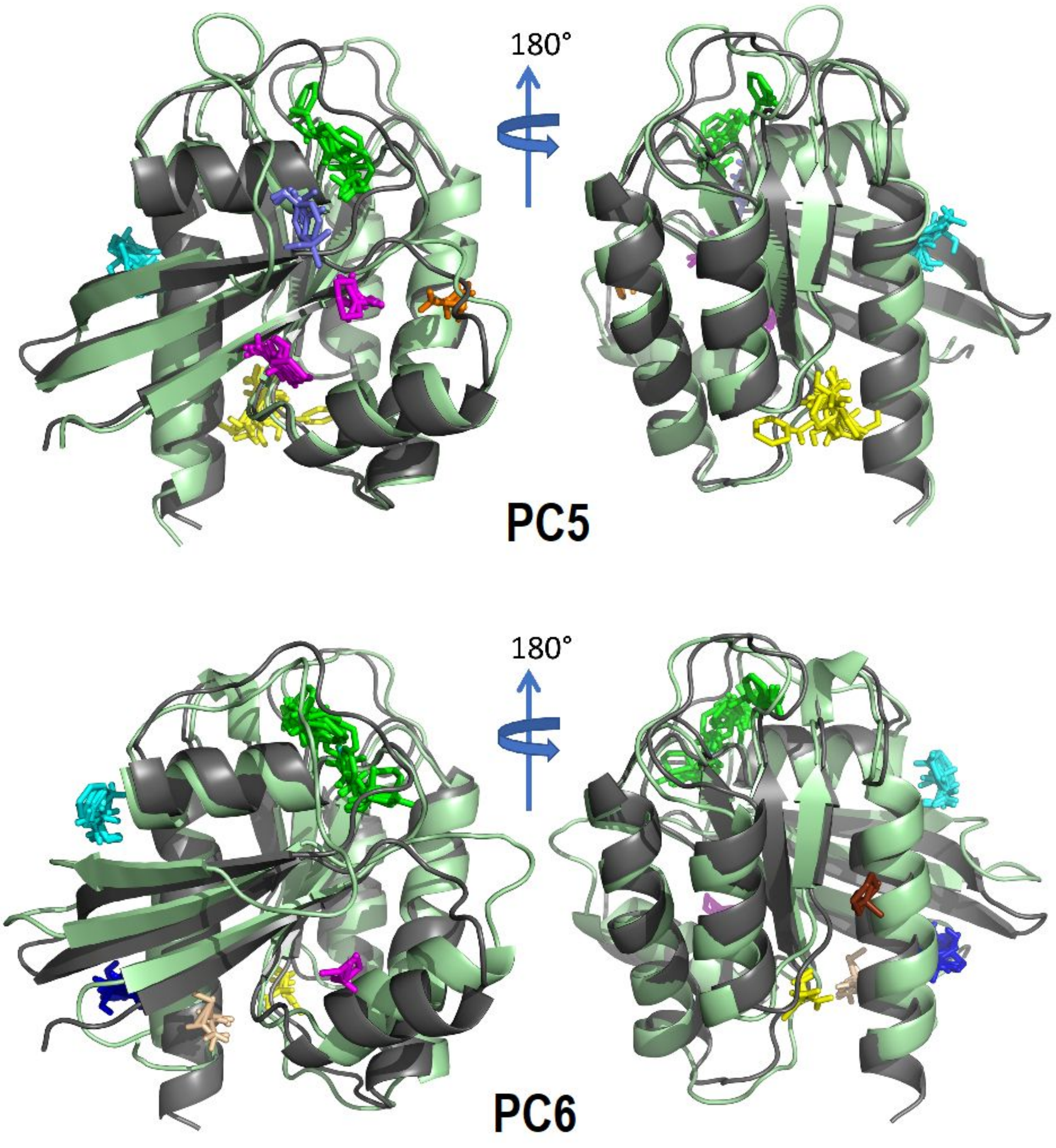

Figure S2. Structures of the 6 representatives (light green) superimposed onto the structure used as starting structure in the present work (pdb id: 4EPX) (gray) based on the Cα of the invariable nucleus. Organic molecules fragments resulting from the FTMAP calculations are explicitly shown.

Table S1. Raw data of the cell proliferation assays using the NIH/3T3 fibroblasts cell line transformed with K-RAS oncogene DNA with the G12V mutation by transfection. Assays were carried out using 5000 cells/well. Cultured cells were seeded and incubated for 24 h. followed by a subsequent incubation for 72 h with or without test compound at 37°C. Cell population was measured by means the absorbance at 490 nm.

Absorbance measurements: 2 experiments/in triplicate

Compound	Absorbance 1	Absorbance 2	Absorbance 3
Control+ Exp#1	2.0703	2.0678	2.0602
Exp#2	1.89	1.8792	1.8745
A55001 50uM	1.9811	1.9747	1.9727
	1.813	1.802	1.7971
A55002 50uM	1.9646	1.9607	1.9579
	1.841	1.8386	1.8335
A55003 50uM	1.2474	1.2478	1.2455
	1.2096	1.2053	1.2017
A55004 50uM	1.4409	1.4346	1.4316
	1.5873	1.5797	1.5719
A55005 50uM	1.8342	1.8277	1.8267
	2.03	2.0258	2.0222
A55006 50uM	1.7762	1.7722	1.7685
	1.9835	1.9699	1.9648
A55007 50uM	1.3151	1.3099	1.3097
	1.4343	1.4291	1.427
vehicle	0.3694	0.3632	0.362
	0.3464	0.3405	0.3446
w/cells @ 24 h	0.448	0.442	0.44
	0.454	0.451	0.446

Compound	Absorption	Average	Background	%Proliferation	%Inhibition
Control+ Exp#1	2.066	1.974	1.527	100%	**0%**
Exp#2	1.881				
A55001 50uM	1.976	1.890	1.443	95%	**5%**
	1.804				
A55002 50uM	1.961	1.899	1.453	95%	**5%**
	1.838				
A55003 50uM	1.247	1.226	0.779	51%	**49%**
	1.206				
A55004 50uM	1.436	1.508	1.061	69%	**31%**
	1.580				
A55005 50uM	1.830	1.928	1.481	97%	**3%**
	2.026				

A55006 50uM	1.772<br>1.973	1.873	1.426	93%	**7%**
A55007 50uM	1.312<br>1.430	1.371	0.924	61%	**39%**
vehicle	0.365<br>0.344	0.354	0.354		
w/cells @ 24 h	0.443<br>0.450	0.447	0.092		

Table S1 (cont.). Raw data of the cell proliferation inhibition assays.

Absorbance measurements: 2 experiments/in triplicate

Compound	Absorbance 1	Absorbance 2	Absorbance 3
Control+	2.1238	2.1053	2.0981
	1.9699	1.9565	1.9505
A55008 50uM	2.0771	2.0558	2.0468
	1.9529	1.943	1.938
A55009 50uM	1.825	1.807	1.8023
	1.6284	1.6118	1.6093
A55010 50uM	2.1009	2.0865	2.0747
	1.8725	1.8535	1.8475
A55011 50uM	1.8763	1.8675	1.8661
	2.0102	1.997	1.9903
A55012 50uM	1.9337	1.9111	1.9086
	2.0076	1.9863	1.9814
A55013 50uM	1.4127	1.3998	1.3954
	1.4454	1.4319	1.4272
vehicle	0.3694	0.3632	0.362
	0.3464	0.3405	0.3446
w/cells @ 24 h	0.481	0.476	0.473
	0.449	0.446	0.446

Compound	Absorption	Average	Background	%Proliferation	% Inhibition
Control+	2.109<br>1.959	2.034	1.572	100%	**0%**
A55008 50uM	2.060<br>1.945	2.002	1.540	98%	**2%**
A55009 50uM	1.811<br>1.617	1.714	1.252	80%	**20%**
A55010 50uM	2.087<br>1.858	1.973	1.511	96%	**4%**
A55011 50uM	1.870<br>1.999	1.935	1.473	94%	**6%**
A55012 50uM	1.918<br>1.992	1.955	1.493	95%	**5%**
A55013 50uM	1.403<br>1.435	1.419	0.957	61%	**39%**
vehicle	0.365<br>0.344	0.354	0.354		
w/cells @ 24 h	0.477<br>0.447	0.462	0.107		

Table S1 (cont.). Raw data of the cell proliferation inhibition assays. In this case, assays were carried out using 4500 cells/well.

Absorbance measurements: 2 experiments/in triplicate

Compound	Absorbance 1	Absorbance 2	Absorbance 3
Control+	1.1338	1.1344	1.1306
	1.2592	1.2541	1.2487
A550014 50uM	1.0848	1.0797	1.0746
	1.0477	1.0439	1.0405
A550015 50uM	1.0866	1.0841	1.081
	1.2161	1.2077	1.2019
A550016 50uM	0.8526	0.8532	0.848
	0.8389	0.838	0.8359
A550017 50uM	1.1216	1.1154	1.1145
	1.266	1.255	1.2502
vehicle	0.3247	0.3222	0.3185
	0.3467	0.3442	0.3388
w/cells @ 24 h	0.4396	0.4329	0.4311
	0.4275	0.4196	0.4165

Compound	Absorption	Average	Background	%Proliferation	% Inhibition
Control+	1.133	1.193	0.766	100%	**0%**
	1.254				
A550014 50uM	1.080	1.062	0.634	83%	**17%**
	1.044				
A550015 50uM	1.084	1.146	0.718	94%	**6%**
	1.209				
A550016 50uM	0.851	0.844	0.417	54%	**46%**
	0.838				
A550017 50uM	1.117	1.187	0.759	99%	**1%**
	1.257				
vehicle	0.322	0.333			
	0.343				
w/cells @ 24h	0.435	0.428	0.095		
	0.421				
